# WHAT ABOUT HOBFOLL’S RESOURCES CONSERVATION MODEL IN A DIGITAL SOCIETY?

**DOI:** 10.1093/geroni/igad104.2212

**Published:** 2023-12-21

**Authors:** Angélique Roquet, Paolo Martinelli, Charikleia Lampraki, Daniela Jopp

**Affiliations:** Lausanne University, Lausanne, Vaud, Switzerland; Lausanne University, Lugaggia, Ticino, Switzerland; Geneva University, Geneve, Geneve, Switzerland; University of Lausanne, Lausanne, Vaud, Switzerland

## Abstract

Internet use has dramatically increased worldwide, with over two-thirds of the world’s population using it, including the elderly population. Technological resources, such as internet use, have been shown to influence psychological variables, such as stress. According to Hobfoll’s theory, stress perception depends on individual’s resources and their changes. While resources and stress are negatively associated, we ignore the role of technological resources on the relationship between personal resources and stress. This study aims at investigating the moderating role of technological resources (internet use) on the relationship between personal resources and stress in young and older adults. A total of 275 young (18-30 years) and 224 older adults (65 years or older) indicated their levels of stress, personal resources changes (i.e., cognitive, social, and self-efficacy resources loss and gain), and internet use. Results showed that the relationship between resource loss, resource gain, and stress in older adults was moderated by their level of internet use. Specifically, older adults who used internet more frequently were less stressed when they experienced both high levels of loss and gain, compared to their counterparts who used less internet in the same conditions. Furthermore, older adults with low resource gain and high resource loss expressed less stress when they used more internet compared to those who had low internet use. These findings highlight the important role of internet use in mitigating stress among older adults experiencing resource loss and gain, emphasizing the potential of digital interventions to promote mental health and well-being in this population.

